# 2-Phenyl­imidazolium acetate

**DOI:** 10.1107/S1600536810004939

**Published:** 2010-02-17

**Authors:** Dao-Cheng Xia, Ji-Huan Yao

**Affiliations:** a Yuncheng University, College of Chemistry, Yuncheng 044000, People’s Republic of China

## Abstract

There are two 2-phenyl­imidazole cations and two acetate anions in the asymmetric unit of the title mol­ecular salt, C_9_H_9_N_2_^+^·C_2_H_3_O_2_^−^. The dihredral angles between the five- and six-membered rings are 5.50 (2) and 6.90 (2)° in the two molecules. The structure is stabilized by N—H⋯O and weak C—H⋯O hydrogen-bonding inter­actions between the cations and anions, resulting in chains propagating in [110].

## Related literature

For related structures, see: Liu *et al.* (2008 ▶); Yang *et al.* (2008 ▶); Xia *et al.* (2009 ▶).
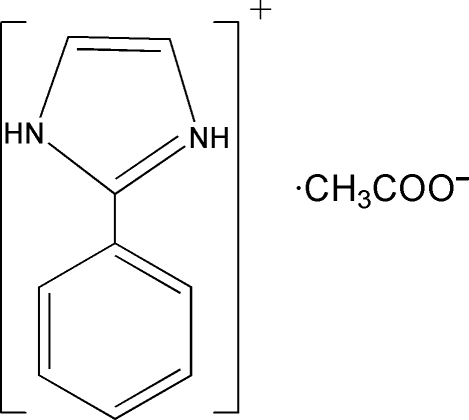


## Experimental

### 

#### Crystal data

C_9_H_9_N_2_^+^·C_2_H_3_O_2_^−^
*M* *_r_* = 204.23
Monoclinic, 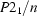

*a* = 10.0320 (6) Å
*b* = 11.0043 (7) Å
*c* = 19.3936 (9) Å
β = 97.982 (5)°
*V* = 2120.2 (2) Å^3^
*Z* = 8
Mo *K*α radiation
μ = 0.09 mm^−1^
*T* = 293 K
0.21 × 0.18 × 0.17 mm

#### Data collection

Oxford Diffraction Gemini R Ultra diffractometer
Absorption correction: multi-scan (*CrysAlis RED*; Oxford Diffraction, 2006 ▶) *T*_min_ = 0.57, *T*_max_ = 0.81
9257 measured reflections
4331 independent reflections
2142 reflections with *I* > 2 σ(*I*)
*R*_int_ = 0.023

#### Refinement

*R*[*F* ^2^ > 2σ(*F* ^2^)] = 0.041
*wR*(*F* ^2^) = 0.114
*S* = 0.82
4331 reflections
272 parameters
H-atom parameters constrained
Δρ_max_ = 0.17 e Å^−3^
Δρ_min_ = −0.16 e Å^−3^

### 

Data collection: *CrysAlis CCD* (Oxford Diffraction, 2006 ▶); cell refinement: *CrysAlis CCD*; data reduction: *CrysAlis RED*; program(s) used to solve structure: *SHELXS97* (Sheldrick, 2008 ▶); program(s) used to refine structure: *SHELXL97* (Sheldrick, 2008 ▶); molecular graphics: *SHELXTL* (Sheldrick, 2008 ▶); software used to prepare material for publication: *SHELXTL*.

## Supplementary Material

Crystal structure: contains datablocks global, I. DOI: 10.1107/S1600536810004939/pv2258sup1.cif

Structure factors: contains datablocks I. DOI: 10.1107/S1600536810004939/pv2258Isup2.hkl

Additional supplementary materials:  crystallographic information; 3D view; checkCIF report

## Figures and Tables

**Table 1:** Hydrogen-bond geometry (Å, °)

*D*—H⋯*A*	*D*—H	H⋯*A*	*D*⋯*A*	*D*—H⋯*A*
N1—H1⋯O1^i^	0.86	1.75	2.606 (2)	172
N2—H2⋯O3	0.86	1.86	2.720 (2)	175
N3—H3⋯O2	0.86	1.81	2.667 (2)	175
N4—H4⋯O4^ii^	0.86	1.76	2.609 (2)	169
C2—H2*A*⋯O3	0.93	2.48	3.374 (2)	161
C6—H6⋯O1^i^	0.93	2.52	3.407 (3)	158
C8—H8⋯O4^iii^	0.93	2.53	3.430 (2)	164
C14—H14⋯O4^ii^	0.93	2.55	3.439 (2)	159
C18—H18⋯O2	0.93	2.41	3.309 (2)	162

Symmetry codes: (i) 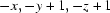; (ii) 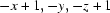; (iii) 
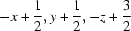
.

**Fig. 1.**
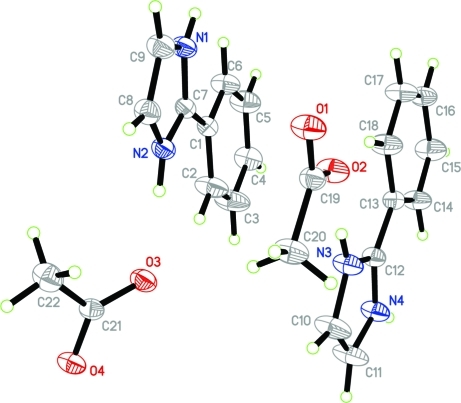
The structure of (I), showing the atomic numbering scheme. Displacement ellipsoids are drawn at the 30% probability level.

## supplementary crystallographic information

### Comment

2-Phenylimidazole has been extensively used to build supramolecular architectures (Liu *et al.*, 2008; Yang *et al.*, 2008). Continuing our research in this important field (Xia *et al.*, 2009), we now report the preparation and crystal structure of the title compound, (I).

There are two 2-phenylimidazole cations and two acetate anions in the asymmetric unit of the title compound (Fig. 1). In the crystal, the cations and anions are linked into chains along [110] by the N—H···O H–bonding interactions (Table 1) thus stabilizing the structure. The structure is further stabilized by rather weak non-classical interactions of the type C—H···O.

### Experimental

A mixture of 2-phenylimidazole (0.5 mmol), CH_3_COOH (0.5 mmol) and H_2_O (30 mmol) was mixed. After one week, colorless crystals of (I) were yielded at room temperature (27% yield).

### Refinement

All H atoms on C and N atoms were positioned geometrically (N—H = 0.86 Å and C—H = 0.93 and 0.96 Å for aryl and methyl H-atoms, respectively) and refined as riding, with U_iso_(H) = 1.5U_eq_(methyl-C atoms) and 1.2U_eq_(the rest of the carrier C/N atoms).

### Crystal data

**Table:**

C_9_H_9_N_2_^+^·C_2_H_3_O_2_^−^	*F*(000) = 864
*M**_r_* = 204.23	*D*_x_ = 1.280 Mg m^−^^3^
Monoclinic, *P*2_1_/*n*	Mo *K*α radiation, λ = 0.71073 Å
Hall symbol: -P 2yn	Cell parameters from 4331 reflections
*a* = 10.0320 (6) Å	θ = 2.1–26.4°
*b* = 11.0043 (7) Å	µ = 0.09 mm^−^^1^
*c* = 19.3936 (9) Å	*T* = 293 K
β = 97.982 (5)°	Block, colorless
*V* = 2120.2 (2) Å^3^	0.21 × 0.18 × 0.17 mm
*Z* = 8	

### Data collection

**Table:**

Oxford Diffraction Gemini R Ultra diffractometer	4331 independent reflections
Radiation source: fine-focus sealed tube	2142 reflections with *I* > 2.o σ(*I*)
graphite	*R*_int_ = 0.023
Detector resolution: 10.0 pixels mm^-1^	θ_max_ = 26.4°, θ_min_ = 2.1°
ω scan	*h* = −12→12
Absorption correction: multi-scan (*CrysAlis RED*; Oxford Diffraction, 2006)	*k* = −9→13
*T*_min_ = 0.57, *T*_max_ = 0.81	*l* = −18→24
9257 measured reflections	

### Refinement

**Table:**

Refinement on *F*^2^	Secondary atom site location: difference Fourier map
Least-squares matrix: full	Hydrogen site location: inferred from neighbouring sites
*R*[*F*^2^ > 2σ(*F*^2^)] = 0.041	H-atom parameters constrained
*wR*(*F*^2^) = 0.114	*w* = 1/[σ^2^(*F*_o_^2^) + (0.0675*P*)^2^] where *P* = (*F*_o_^2^ + 2*F*_c_^2^)/3
*S* = 0.82	(Δ/σ)_max_ = 0.001
4331 reflections	Δρ_max_ = 0.17 e Å^−^^3^
272 parameters	Δρ_min_ = −0.16 e Å^−^^3^
0 restraints	Extinction correction: *SHELXL*, Fc^*^=kFc[1+0.001xFc^2^λ^3^/sin(2θ)]^-1/4^
Primary atom site location: structure-invariant direct methods	Extinction coefficient: 0.0175 (15)

### Special details

**Table:**

Geometry. All esds (except the esd in the dihedral angle between two l.s. planes) are estimated using the full covariance matrix. The cell esds are taken into account individually in the estimation of esds in distances, angles and torsion angles; correlations between esds in cell parameters are only used when they are defined by crystal symmetry. An approximate (isotropic) treatment of cell esds is used for estimating esds involving l.s. planes.
Refinement. Refinement of *F*^2^ against ALL reflections. The weighted *R*-factor wR and goodness of fit *S* are based on *F*^2^, conventional *R*-factors *R* are based on *F*, with *F* set to zero for negative *F*^2^. The threshold expression of *F*^2^ > σ(*F*^2^) is used only for calculating *R*-factors(gt) etc. and is not relevant to the choice of reflections for refinement. *R*-factors based on *F*^2^ are statistically about twice as large as those based on *F*, and *R*- factors based on ALL data will be even larger.

### Fractional atomic coordinates and isotropic or equivalent isotropic displacement parameters (Å^2^)

**Table:**

	*x*	*y*	*z*	*U*_iso_*/*U*_eq_	
C1	0.03640 (17)	0.18563 (16)	0.46297 (8)	0.0512 (5)	
C2	0.1416 (2)	0.1044 (2)	0.46747 (10)	0.0877 (7)	
H2A	0.2015	0.0994	0.5085	0.105*	
C3	0.1595 (3)	0.0308 (2)	0.41253 (11)	0.1005 (9)	
H3A	0.2313	−0.0233	0.4167	0.121*	
C4	0.0733 (2)	0.03613 (19)	0.35197 (10)	0.0788 (6)	
H4A	0.0856	−0.0139	0.3147	0.095*	
C5	−0.0309 (2)	0.1154 (2)	0.34671 (11)	0.0829 (7)	
H5	−0.0904	0.1196	0.3055	0.100*	
C6	−0.0498 (2)	0.18957 (19)	0.40128 (10)	0.0732 (6)	
H6	−0.1220	0.2434	0.3965	0.088*	
C7	0.01993 (17)	0.26654 (16)	0.52036 (8)	0.0495 (4)	
C8	0.05331 (19)	0.36114 (18)	0.62119 (9)	0.0629 (5)	
H8	0.0891	0.3829	0.6663	0.075*	
C9	−0.0501 (2)	0.41439 (18)	0.58166 (9)	0.0683 (6)	
H9	−0.0992	0.4805	0.5943	0.082*	
C10	0.5548 (2)	0.2029 (2)	0.49467 (11)	0.0949 (8)	
H10	0.5801	0.2131	0.5423	0.114*	
C11	0.6097 (2)	0.1266 (2)	0.45397 (11)	0.0956 (8)	
H11	0.6807	0.0737	0.4679	0.115*	
C12	0.44891 (18)	0.22354 (17)	0.38869 (9)	0.0559 (5)	
C13	0.35306 (17)	0.26213 (15)	0.32912 (8)	0.0504 (4)	
C14	0.3567 (2)	0.2150 (2)	0.26411 (10)	0.0761 (6)	
H14	0.4247	0.1609	0.2570	0.091*	
C15	0.2611 (2)	0.2466 (2)	0.20906 (10)	0.0883 (7)	
H15	0.2648	0.2128	0.1654	0.106*	
C16	0.1615 (2)	0.32649 (19)	0.21777 (10)	0.0710 (6)	
H16	0.0972	0.3478	0.1805	0.085*	
C17	0.1577 (2)	0.37441 (19)	0.28156 (10)	0.0806 (7)	
H17	0.0901	0.4292	0.2882	0.097*	
C18	0.2532 (2)	0.34293 (19)	0.33701 (10)	0.0768 (6)	
H18	0.2492	0.3774	0.3805	0.092*	
C19	0.32986 (18)	0.48865 (18)	0.55858 (9)	0.0571 (5)	
C20	0.4364 (2)	0.4355 (2)	0.61201 (10)	0.0811 (7)	
H20A	0.4395	0.4798	0.6548	0.122*	
H20B	0.5222	0.4408	0.5956	0.122*	
H20C	0.4157	0.3518	0.6198	0.122*	
C21	0.30718 (17)	0.07402 (17)	0.69394 (8)	0.0518 (5)	
C22	0.2379 (2)	0.14122 (19)	0.74594 (10)	0.0787 (6)	
H22A	0.2543	0.1006	0.7901	0.118*	
H22B	0.1429	0.1435	0.7303	0.118*	
H22C	0.2722	0.2227	0.7508	0.118*	
N1	−0.07063 (15)	0.35460 (14)	0.51965 (7)	0.0600 (4)	
H1	−0.1323	0.3712	0.4856	0.072*	
N2	0.09625 (14)	0.26903 (13)	0.58294 (6)	0.0540 (4)	
H2	0.1616	0.2203	0.5966	0.065*	
N3	0.45439 (16)	0.26380 (15)	0.45407 (7)	0.0711 (5)	
H3	0.4034	0.3187	0.4681	0.085*	
N4	0.54380 (16)	0.13943 (14)	0.38802 (7)	0.0701 (5)	
H4	0.5608	0.0997	0.3520	0.084*	
O1	0.26407 (14)	0.57803 (13)	0.57651 (6)	0.0737 (4)	
O2	0.31032 (13)	0.44328 (12)	0.49935 (6)	0.0750 (4)	
O3	0.29723 (12)	0.11352 (12)	0.63317 (6)	0.0673 (4)	
O4	0.37241 (12)	−0.02072 (11)	0.71421 (5)	0.0642 (4)	

### Atomic displacement parameters (Å^2^)

**Table:**

	*U*^11^	*U*^22^	*U*^33^	*U*^12^	*U*^13^	*U*^23^
C1	0.0531 (11)	0.0512 (11)	0.0485 (10)	0.0027 (9)	0.0046 (8)	0.0053 (8)
C2	0.1034 (17)	0.0981 (17)	0.0551 (12)	0.0501 (14)	−0.0114 (11)	−0.0115 (12)
C3	0.118 (2)	0.1063 (19)	0.0703 (14)	0.0594 (16)	−0.0112 (13)	−0.0202 (13)
C4	0.0918 (16)	0.0745 (15)	0.0666 (13)	0.0126 (13)	−0.0009 (11)	−0.0162 (11)
C5	0.0869 (16)	0.0860 (16)	0.0676 (13)	0.0161 (13)	−0.0186 (11)	−0.0186 (12)
C6	0.0732 (14)	0.0746 (14)	0.0667 (12)	0.0202 (11)	−0.0086 (10)	−0.0083 (11)
C7	0.0524 (11)	0.0502 (11)	0.0460 (9)	0.0071 (9)	0.0076 (8)	0.0084 (8)
C8	0.0749 (13)	0.0677 (14)	0.0461 (9)	0.0115 (11)	0.0084 (9)	−0.0007 (10)
C9	0.0818 (14)	0.0701 (14)	0.0532 (11)	0.0242 (11)	0.0107 (10)	−0.0026 (10)
C10	0.1201 (19)	0.1024 (19)	0.0555 (12)	0.0555 (16)	−0.0113 (12)	−0.0029 (12)
C11	0.1139 (19)	0.1034 (19)	0.0626 (13)	0.0610 (16)	−0.0123 (12)	0.0016 (13)
C12	0.0571 (12)	0.0536 (12)	0.0560 (11)	0.0114 (9)	0.0041 (8)	0.0002 (9)
C13	0.0475 (10)	0.0484 (11)	0.0533 (10)	0.0040 (9)	0.0002 (8)	−0.0026 (8)
C14	0.0737 (14)	0.0895 (16)	0.0623 (12)	0.0335 (12)	−0.0003 (10)	−0.0080 (11)
C15	0.0917 (17)	0.1143 (19)	0.0546 (12)	0.0340 (15)	−0.0044 (11)	−0.0150 (12)
C16	0.0619 (13)	0.0847 (15)	0.0610 (12)	0.0135 (11)	−0.0106 (10)	−0.0015 (11)
C17	0.0730 (14)	0.0889 (16)	0.0731 (13)	0.0335 (12)	−0.0144 (11)	−0.0154 (12)
C18	0.0798 (14)	0.0843 (15)	0.0598 (11)	0.0301 (12)	−0.0129 (10)	−0.0202 (11)
C19	0.0555 (11)	0.0621 (13)	0.0525 (11)	0.0081 (10)	0.0036 (9)	0.0016 (10)
C20	0.0828 (15)	0.0834 (16)	0.0703 (13)	0.0202 (12)	−0.0138 (11)	0.0009 (11)
C21	0.0486 (10)	0.0578 (12)	0.0454 (10)	0.0007 (9)	−0.0057 (8)	−0.0034 (9)
C22	0.1009 (16)	0.0776 (15)	0.0563 (11)	0.0177 (13)	0.0065 (10)	−0.0068 (11)
N1	0.0645 (10)	0.0650 (10)	0.0488 (9)	0.0173 (8)	0.0021 (7)	0.0056 (8)
N2	0.0579 (9)	0.0567 (10)	0.0467 (8)	0.0109 (7)	0.0045 (7)	0.0057 (7)
N3	0.0800 (11)	0.0759 (12)	0.0542 (9)	0.0321 (9)	−0.0024 (8)	−0.0026 (8)
N4	0.0808 (11)	0.0694 (11)	0.0567 (9)	0.0303 (9)	−0.0022 (8)	−0.0028 (8)
O1	0.0818 (9)	0.0766 (10)	0.0590 (8)	0.0280 (8)	−0.0030 (6)	−0.0086 (7)
O2	0.0823 (10)	0.0845 (10)	0.0550 (8)	0.0301 (8)	−0.0018 (7)	−0.0102 (7)
O3	0.0727 (9)	0.0785 (10)	0.0495 (7)	0.0216 (7)	0.0042 (6)	0.0081 (6)
O4	0.0705 (9)	0.0657 (9)	0.0519 (7)	0.0191 (7)	−0.0074 (6)	0.0006 (6)

### Geometric parameters (Å, °)

**Table:**

C1—C2	1.376 (2)	C13—C18	1.364 (2)
C1—C6	1.376 (2)	C13—C14	1.369 (2)
C1—C7	1.453 (2)	C14—C15	1.377 (2)
C2—C3	1.370 (3)	C14—H14	0.9300
C2—H2A	0.9300	C15—C16	1.359 (3)
C3—C4	1.360 (3)	C15—H15	0.9300
C3—H3A	0.9300	C16—C17	1.350 (3)
C4—C5	1.354 (3)	C16—H16	0.9300
C4—H4A	0.9300	C17—C18	1.381 (2)
C5—C6	1.370 (3)	C17—H17	0.9300
C5—H5	0.9300	C18—H18	0.9300
C6—H6	0.9300	C19—O2	1.2429 (19)
C7—N1	1.327 (2)	C19—O1	1.260 (2)
C7—N2	1.3425 (19)	C19—C20	1.500 (2)
C8—C9	1.337 (2)	C20—H20A	0.9600
C8—N2	1.361 (2)	C20—H20B	0.9600
C8—H8	0.9300	C20—H20C	0.9600
C9—N1	1.361 (2)	C21—O3	1.2470 (19)
C9—H9	0.9300	C21—O4	1.264 (2)
C10—C11	1.323 (3)	C21—C22	1.497 (3)
C10—N3	1.366 (2)	C22—H22A	0.9600
C10—H10	0.9300	C22—H22B	0.9600
C11—N4	1.363 (2)	C22—H22C	0.9600
C11—H11	0.9300	N1—H1	0.8600
C12—N4	1.329 (2)	N2—H2	0.8600
C12—N3	1.337 (2)	N3—H3	0.8600
C12—C13	1.460 (2)	N4—H4	0.8600
			
C2—C1—C6	117.35 (17)	C16—C15—C14	120.78 (19)
C2—C1—C7	121.14 (15)	C16—C15—H15	119.6
C6—C1—C7	121.48 (16)	C14—C15—H15	119.6
C3—C2—C1	121.11 (17)	C17—C16—C15	118.79 (17)
C3—C2—H2A	119.4	C17—C16—H16	120.6
C1—C2—H2A	119.4	C15—C16—H16	120.6
C4—C3—C2	120.7 (2)	C16—C17—C18	120.70 (19)
C4—C3—H3A	119.7	C16—C17—H17	119.6
C2—C3—H3A	119.7	C18—C17—H17	119.6
C5—C4—C3	119.0 (2)	C13—C18—C17	121.13 (18)
C5—C4—H4A	120.5	C13—C18—H18	119.4
C3—C4—H4A	120.5	C17—C18—H18	119.4
C4—C5—C6	120.92 (18)	O2—C19—O1	123.17 (15)
C4—C5—H5	119.5	O2—C19—C20	119.18 (18)
C6—C5—H5	119.5	O1—C19—C20	117.65 (16)
C5—C6—C1	120.98 (19)	C19—C20—H20A	109.5
C5—C6—H6	119.5	C19—C20—H20B	109.5
C1—C6—H6	119.5	H20A—C20—H20B	109.5
N1—C7—N2	107.34 (15)	C19—C20—H20C	109.5
N1—C7—C1	126.18 (14)	H20A—C20—H20C	109.5
N2—C7—C1	126.44 (15)	H20B—C20—H20C	109.5
C9—C8—N2	107.04 (15)	O3—C21—O4	123.46 (17)
C9—C8—H8	126.5	O3—C21—C22	118.73 (16)
N2—C8—H8	126.5	O4—C21—C22	117.81 (16)
C8—C9—N1	107.77 (17)	C21—C22—H22A	109.5
C8—C9—H9	126.1	C21—C22—H22B	109.5
N1—C9—H9	126.1	H22A—C22—H22B	109.5
C11—C10—N3	107.58 (17)	C21—C22—H22C	109.5
C11—C10—H10	126.2	H22A—C22—H22C	109.5
N3—C10—H10	126.2	H22B—C22—H22C	109.5
C10—C11—N4	107.85 (17)	C7—N1—C9	108.93 (14)
C10—C11—H11	126.1	C7—N1—H1	125.5
N4—C11—H11	126.1	C9—N1—H1	125.5
N4—C12—N3	107.75 (14)	C7—N2—C8	108.91 (14)
N4—C12—C13	126.08 (16)	C7—N2—H2	125.5
N3—C12—C13	126.14 (17)	C8—N2—H2	125.5
C18—C13—C14	117.67 (15)	C12—N3—C10	108.30 (16)
C18—C13—C12	120.96 (16)	C12—N3—H3	125.9
C14—C13—C12	121.32 (17)	C10—N3—H3	125.9
C13—C14—C15	120.93 (19)	C12—N4—C11	108.52 (16)
C13—C14—H14	119.5	C12—N4—H4	125.7
C15—C14—H14	119.5	C11—N4—H4	125.7

### Hydrogen-bond geometry (Å, °)

**Table:**

*D*—H···*A*	*D*—H	H···*A*	*D*···*A*	*D*—H···*A*
N1—H1···O1^i^	0.86	1.75	2.606 (2)	172
N2—H2···O3	0.86	1.86	2.720 (2)	175
N3—H3···O2	0.86	1.81	2.667 (2)	175
N4—H4···O4^ii^	0.86	1.76	2.609 (2)	169
C2—H2A···O3	0.93	2.48	3.374 (2)	161
C6—H6···O1^i^	0.93	2.52	3.407 (3)	158
C8—H8···O4^iii^	0.93	2.53	3.430 (2)	164
C14—H14···O4^ii^	0.93	2.55	3.439 (2)	159
C18—H18···O2	0.93	2.41	3.309 (2)	162

Symmetry codes: (i) −*x*, −*y*+1, −*z*+1; (ii) −*x*+1, −*y*, −*z*+1; (iii) −*x*+1/2, *y*+1/2, −*z*+3/2.
